# Supporting One Health for Pandemic Prevention: The Need for Ethical Innovation

**DOI:** 10.1007/s11673-023-10264-5

**Published:** 2023-06-02

**Authors:** Elena R. Diller, Laura Williamson

**Affiliations:** Center for Bioethics and Health Policy, Institute of Public and Preventive Health, Augusta University, 1120 15th St., Augusta, GA 30912 USA

**Keywords:** Bioethics, One Health, Pandemic prevention, COVID-19, Global health, Animal health, Environmental health

## Abstract

Bioethics is a field in which innovation is required to help prevent and respond to zoonotic diseases with the potential to cause epidemics and pandemics. Some of the developments necessary to fight pandemics, such as COVID-19 vaccines, require public debate on the benefits and risks of individual choice versus responsibility to society. While these debates are necessary, a more fundamental ethical innovation to rebalance human, animal, and environmental interests is also needed. One Health (OH) can be characterized as a strategy that recognizes and promotes the synergy between human, animal, and environmental health. Yet, despite the recognition that these entities are interdependent, there is a pronounced inequality in the power relations between human, non-human animal, and the environmental interests which threatens the well-being of all. Until OH can ensure the moral status of animals and the environment and thereby the equal consideration of these interests, it will struggle to protect non-human interests and, as a result, human health. To create a sustainable health system requires a renewed concept of justice that is ecocentric in nature and an application of OH that is flexible and responsive to different ethical interests (e.g., person-centred care and physician responsibilities). Ultimately, to save themselves, humans must now think beyond themselves. Bioethics must assume a key role in supporting the developments required to create and maintain relationships able to sustain environmental and human health.

## Introduction

The COVID-19 pandemic has presented humanity with practical and ethical challenges. For multiple reasons, public health messaging has failed to engage many people in liberal democracies (Wilkin 2020). This shortcoming has been partly responsible for millions of deaths. It has also highlighted the long-standing tension between individual choice and responsibility to society (Bourgeois, Harrell, and Stephenson 2020). As the pandemic is now considered an endemic (Spencer 2022), public health messaging has continued to urge people to “follow the science” and abide by recommendations to mask, vaccinate, and quarantine. While understandable as a fire-fighting measure, there has been little public conversation and debate about the entrenched habits by which humans put themselves at risk for future pandemics. It is also important to examine the importance of a more deep-rooted preventive approach. Such a strategy is important because SARS-CoV-2 virus is the third zoonotic coronavirus to appear in the past decade (Nicol 2021). Research shows that human practices including deforestation, the hunting of wildlife and consumption of its meat, drive the spread of zoonotic infection (Gibb et al. 2020). The examination of such issues raises ethical and not only empirical concerns. However, traditional, Western Bioethics has largely focused on human interests to the exclusion animal and environmental concerns. It is often individually, rather than socially focused as a result (Williamson 2014). The systematic development of public health ethics since around 2000 has helped to highlight the importance of focusing on the community or social dimensions of health (Callahan and Jennings 2002); but, as we will argue, this important work has not yet shifted to meaningfully include the interests of non-human animals and the environment. Rather, ethical innovation is required to inform health debates about human health issues intrinsically tied to animals and the environment, such as the COVID-19 pandemic and other vector borne diseases.

The One Health (OH) approach recognizes and prioritizes the interdependent health of humans, non-human animals, and the environment (Coghlan and Coghlan 2018; Lerner and Berg 2017), and so it has been suggested as a possible approach to pandemic prevention (Arshad et al. 2021; Garcia Pinillos 2021). OH principles (i.e., human health is dependent upon ecosystem health, which requires the mutualism of humans, non-human animals, and the environment) have previously been applied to complex, worldwide health issues, including zoonotic disease control, animal agriculture, and land deforestation (Zinsstag et al. 2011). Instead of sectoral silos, OH encourages transdisciplinary cooperation between fields, such as veterinary medicine, ecology, environmental sciences, public health, and clinical medicine, to incorporate their respective approaches when solving global health issues (Lerner and Berg 2017; Zinsstag et al. 2011). OH has become increasingly popular in the past several decades, as major health agencies have incorporated the heuristic into their policies (Coghlan and Coghlan 2018). As the World Health Organization Director, General Tedors Adhanom Ghebreyesus, recently said during the 27th Tripartite Annual Executive Committee Meeting World Organization for Animal Health in February 2021, “We can only prevent future pandemics with an integrated One Health approach to public health, animal health and the environment we share” (United Nations 2021, ¶2).

In this paper, we explain some challenges facing the OH approach. Namely, that the principles and values that dominate health debates are often informed by traditional bioethics. Traditional bioethics fails to support the OH initiative because it is anthropocentric and individually focused. Although bioethics continues to become more responsive to social and public health challenges, we suggest the individual human focus of its initial iteration remains dominant, making it incompatible with the OH approach. We then move on to argue that OH must develop a new ethical framework which emphasizes ecocentric justice and interdependence and which ensures the protection of the moral statuses of animals and the environment.^1^

## Limitations of One Health in Pandemic Prevention

OH has already inspired current COVID-19 pandemic mitigation strategies. For example, the CDC developed surveillance and reporting infrastructure to help local, state, and federal public health systems capture important laboratory and epidemiologic data on cases of SARS-CoV-2 in animals linked to people diagnosed with COVID-19 (CDC 2021). On a policy level, China has revised its Wild Animal Conservation Law since the pandemic started with many of the revisions supporting the principles of OH, including the importance of biodiversity (Fang and Song 2021). However, the pandemic has also stimulated conversation about the shortcomings of OH applied to the COVID-19 pandemic (de Garine-Wichatitsky et al. 2020; Ruckert et al. 2020; Schmiege et al. 2020). There is important, though disparate, ethical work on the theoretical foundations of OH (Johnson and Degeling 2019; van Herten, Bovenkerk, and Verweij 2018; Verweij and Bovenkerk 2016). From these works come an important critique of the OH approach, namely, that it has focused on its potential to highlight ethical dilemmas without providing guidance on how to address them (van Herten, Bovenkerk, and Verweij 2018). In its broadest sense, OH does not specify a particular ethical framework in which decisions should be made (Capps and Lederman 2015; Johnson and Degeling 2019). Nor does employing the OH framework guarantee that tradeoffs can be avoided (Verweij and Bovenkerk 2016). The need for an ethical framework within OH is particularly acute when proposed solutions prioritize the health of one group over another. For example, in November 2020, the Danish government ordered the culling of 17 million disease-free minks as a precaution to protect the COVID-19 vaccine (Frutos and Devaux 2020). Because there is no underlying framework defining the moral status of non-human animals and the environment (Capps and Lederman 2015; Johnson and Degeling 2019), the OH approach leaves room for interpretation of when and how to prioritize human, non-human animal, and environmental interests (Lysaght et al. 2017; van Herten, Bovenkerk, and Verweij 2018).

It is not surprising then that OH has been deemed anthropocentric. OH has traditionally been employed only when human health is threatened (Coghlan and Coghlan 2018; Kamenshchikova et al. 2019; Lerner and Berg 2017), thereby demonstrating its fundamental lack of concern for the well-being and health of non-human animals and the environment outside of their connection to humans. One conception of OH is that it assumes its role within the overlapping edges of three pre-existing, separate spaces (humans, non-human animals, and the environment). This makes the approach susceptible to “binary thinking which creates hierarchies and boundaries between humans and non-humans, beings and the environment and diseased and healthy bodies” (Davis and Sharp 2020, 3). This conception is supported by a mixed-methods study exploring the perceptions of OH among zoonotic disease experts in Singapore. The study found that experts ranked impacts on human health as a higher priority than impacts on non-human animal health (Lysaght et al. 2017).

Although we acknowledge that at times the most ethical solution for a given conflict may inadvertently harm one group in seeking to promote the greater good, as we argue in the coming section on justice, the overall burdens endured by each group should be in proportion to the respective benefits and harm that group poses to others. However, humans have yet to master a distributive justice that transcends our own interests. There is no doubt that if the shortsighted needs of humans are continually prioritized over the interests of non-human animals and the environment, humans will expedite the destruction of the planet and its natural resources, resulting in loss of human life (IPBES 2019; Plumer 2019). Without an ethical framework which actively and sharply curtails the power humans demonstrate over non-human nature, OH will ultimately fail to achieve its mission to protect the health of any species at all, humans included.

## Traditional Bioethics and One Health

OH has merged the health and well-being of humans, non-human animals, and the environment without resolving the anthropocentrism or individualism underlying previous and current biomedical ethical frameworks. Although bioethics has long been conceived as a connection between medicine and the environment, the ethics of these two fields have grown apart in the last half century (Lecaros 2013; Lee 2017). This is because contemporary biomedical ethics has largely concerned itself with clinical and research ethics, both of which centre upon the well-being of individual humans. Harms to animals and the environment caused by medical practice or research, such as medical waste pollution, are easily justified and further normalized because they are deemed necessary to promote human health (Ferguson 2021). There are popular methods used to mitigate these harms, such as the 3 Rs of animal research—replacing animals, reducing the number of animals in studies, and refining procedures in minimize pain—coined by William Russell and Rex Burch (Russell and Burch 1959). However, biomedical ethical frameworks aiming to improve animal and environmental welfare within clinical and laboratory settings are fundamentally no different than those which ignore non-human nature altogether. Although their work inspired improvements in laboratory animal welfare, Russell and Burch propagated the idea that human health is dependent upon the sacrifice of non-human animals and that this sacrifice can be justified so long as the harms to animals are minimized. The “final” harm, of course, is not minimized as death for lab animals comes regardless of their well-being in life.

The term “narrow bioethics” has been used to describe how clinical medical ethics and research ethics exploit non-human animals and the environment to promote human health (Ferguson 2021). Russell and Burch’s framework is an example of how traditional, “ethical” science (i.e., narrow bioethics) has operated based on the assumption that humans are of the highest moral status (Zurlo, Rudacille, and Goldberg 1996). As noted above, public health ethics is a more recent development within ethical debates on health. It is inherently socially focused, thereby leading some scholars to suggest the development of public health ethics as a bridge between the anthropocentric nature of contemporary bioethics and broader social and environmental priorities (Kessell and Stephens 2011). Lee argues that public health, grounded in its concern for the community, requires individual and environmental health be protected to promote the health and well-being of the public (Lee 2017). The authors of the aforementioned Singapore study discuss how an emphasis on justice in OH policymaking may improve the distribution of resources, benefits, and burdens among humans, non-human animals, and the environment (Lysaght et al. 2017). From this it appears that OH could be served well by public health ethics because of its commitment to justice and eradicating health inequality.

However, there are two reasons why public health ethics stops short of being the ideal ethical framework for OH. First, public health ethics neglects the non-instrumental value of non-human animals and the environment, making its focus as narrow as traditional bioethics (Ferguson 2021). Although Degeling et al. (2016) have argued that OH is consistent with health as a universal, shared good, and for this reason OH could be consistent with the public health agenda, public health ethics has yet to define the moral status of animals or the environment. Arguments regarding distribution of benefits and harms require normative evaluations. Without a clearly outlined position on moral status of these non-human entities, public health ethics lacks the foundation required to give all parties involved their due ethical consideration, which we believe is a minimal requirement for any approach to OH. Second, while public health ethics’ commitment to justice may increase the protection of vulnerable groups or interests, including non-human animals and the environment, OH cannot solely rely upon current conceptions of justice to ensure that the interests of non-human animals and the environment are considered equal to human interests. This is because contemporary accounts of justice tend to follow a core principle which is problematic for non-human animals and the environment, namely, that individuals should be treated equally unless there are differences between them relevant to the situation at hand (Velasquez et al. 2014). Greater ethical innovation is required, fundamental to such change is the need to reconsider the concept of justice and how it weighs different, yet inherently connected interests.

Of note, there are modifications to traditional bioethics which attempt to rectify its anthropocentrism. For example, the post humanist approach to public health expands the focus of concern to include non-human entities, including animals, the environment, space, and material objects. These approaches run parallel to OH; for example, post humanism is consistent with a definition of health as a shared concept upon which multiple entities depend and impact. Yet, in addition to lacking discourse on moral status, they also tend to remain anthropocentric in their concern, focusing on the benefits to humans of non-human entities. As Cohn and Lynch write, “… posthuman perspectives are not about leaving what is human behind, but in fact the opposite—exploring what being human means in relation to what might be deemed as not human” (2017). While these endeavours are meaningful, they alone do not get us closer to a sustainable ethic that considers the interests of humans, non-human animals, and the environment alike.

## Justice in One Health

While the arguments for or against the inclusion of animals in justice merit their own discussion,^2^ we assert that a justice compatible with sustainable global health must consider animal and environmental interests. We argue a distributive justice is most consistent with One Health, as it is a strategy employed by institutions that enact change on a large-scale. For distributive justice to be fair, we stipulate the overall burdens endured by each group should be in proportion to the respective benefits and harm that group poses to others. Yet, justice has traditionally focused on weighing different human interests, rather than those of humans, non-human animals, and the environment. Thus, to use justice as a core principle of OH in today’s world requires the development and/or popularization of an account of justice that takes seriously the equal consideration of the interests of inherently different parties with varying degrees of power to protect those interests. Justice needs to be equipped to work with a far greater array of diversity than it is currently accustomed, and those executing justice must be equipped to do so from a neutral position.^3^

While the original Rawlsian contractarian theory, the veil of ignorance, excludes animals, it is a particularly useful thought experiment in imagining a non-anthropocentric form of justice (Rawls 1971). Hilden offers several convincing reasons why non-human animals should be included in original position, including that species is not a relevant difference nor is it an ethical argument for who deserves moral status (Hilden 2007). If representatives behind the veil of ignorance were to consider that they may become a cow in a slaughterhouse, a rat in a cosmetic testing laboratory or a river filled with human waste, how would their views of justice change? A solution to culling, for example, would be more likely to respect the principles of OH if those choosing the solution considered themselves behind a veil of ignorance which included both the cullers and the culled (Lederman 2016).^4^

What if the representatives behind the veil further understood that their fate was not just dependent upon the well-being of their own species but the well-being of *every* species? An account of justice for OH must respect that each party has the potential to harm and benefit the others; that is to say, each party is crucial to the survival of the other parties, and because of that, everyone is really part of the same system of wellness. Thus, OH needs an ethical framework that not only considers the interdependence of different interests (e.g., human, non-human animals, and the environment) but promotes the health of the system over the health of any one group. While a justice for OH must be comparative in that there is equal consideration of different interests, it must also accept that to some degree, the health of the system must be prioritized to ensure the common good of everyone.

Another possible ethical approach to protecting all parties involved in OH (e.g., humans, non-human animals, and the environment) is ecojustice. This approach posits that at times we must supersede anthropocentric forms of justice, as seen in bioethics and public health ethics, to protect the natural world upon which all living and non-living things rely (Des Jardins 2013; Washington et al. 2018). Ecojustice prioritizes the ecosystem, acknowledging that doing so may mean sacrificing individual elements of the ecosystem, either living or non-living, to preserve the health of the system (Des Jardins 2013; Cryer et al. 2020; Washington et al. 2018). Ecojustice is suitable as a core ethical value of OH because it respects the mutualism between humans, non-human animals, and the environment.

Ecojustice, however, will only be realized when humans believe just as strongly in ecocentrism as they currently do in individualism and anthropocentrism. Ecocentrism is the philosophy that the ecosystem is intrinsically valuable. It is a philosophy grounded within extensive scientific evidence that each species is evolutionarily benefited by others (Washington et al. 2018). To make decisions that merit the equal consideration of extremely different, yet inherently connected interests, humans must believe that these other parties (e.g., non-human animals and the environment) are as crucial to the ecosystem as they believe themselves to be. As Washington et al., write, “the fear of giving nature an equal moral footing … is a major obstacle to reaching a viable concept of justice that encompasses both humans and nature, and hence achieving a holistic conservation strategy for planet Earth” (370).

An expanded version of the veil of ignorance and ecojustice are two different ethical approaches that can be utilized to support OH problem solving. The veil of ignorance helps people thinking about justice to adopt a process that makes their assessments more inclusive of all interests; and ecojustice substantively challenges the narrow individualism that continues to blight traditional bioethics. Together these approaches help OH users to consider the interests of non-human animals and the environment more equally to those of humans. That is, both frameworks facilitate the redistribution of power to include those who are ultimately excluded from participating in decision-making. In this sense, humans utilizing OH will better recognize, respect, and protect the health interests of non-human animals and the environment.

To support such approaches, however, arguably requires even more fundamental ethical innovation and a shift from using an anthropocentric, humanist lens towards one that is posthuman (McLaughlin 2019). It has been argued, for example, that anthropocentricism still governs “the supposedly free ‘choices’ being made in neoliberal education systems” around the teaching of environmental education (Kopnina and Cherniak 2015). Yet, ethical debate on non-human animal, human, and environmental interests requires a philosophical foundation that consistently appreciates that the moral universe does not require prioritizing human beings. The implications of this for bioethics are profound as its foundations are challenged and portrayed as inadequate for contemporary health challenges. For bioethics, a field that has consistently utilized an anthropocentric lens to justify the use of animals in research and minimize their consideration in global health ethics debates (Benatar 2011), the challenges of seriously pursuing a commitment to OH require fundamental change.

## Conclusion: Thinking Across Health Systems

Given the degree of ethical innovation required to significantly support OH to help address the health challenges raised by the COVID-19 pandemic, it is important bioethics dedicates substantial attention to considering its future. We contend that this future requires either far reaching change or an acknowledgement that the field continues support of the status quo—a position that constitutes, not just a lack of moral imagination but a catastrophic ethical failure to support the type of strategies needed to protect human, non-human animal, and environmental health. For example, it is important to acknowledge that the development of an ethic to support OH would see it conflict with other well-established ethical fields like clinical and research ethics. Those currently working in these fields—directors and members of Institution Review Boards (IRBs) and physicians—have responsibilities to help reimagine bioethics to help it meet contemporary health challenges. The American Medical Association not only recognizes climate change as a medical emergency but also charges physicians with the duty to protect public health as it relates to climate change, as well as teach patients about environmentally sustainable practices (AMA 2014).

Similarly, person and citizen engagement are widely seen as critical to supporting individual and public health (Williamson 2014). Such engagement efforts will also need to extend their focus to consider the responsibilities people have to balance different interests. This will require not only ensuring those involved have accurate information but support to consider ethical issues beyond the scope of traditional biomedical ethics and its individual focus. This necessitates a step-change in the content of ethics education for professionals; and the cultivation of increased ethics debate in the public forum. Such work must show respect for human life, while also promoting equity through clear communication about the extent to which humans must think beyond themselves to save themselves. While the development of bioethics has long been based upon the idea that individual choice and autonomy are sufficient for well-being, the next stage of the field’s development requires it take a more critical stance towards this status quo if it is to meaningfully support health.

Ethical frameworks (such as the veil of ignorance and ecojustice) can help to promote and protect the interdependence between different types of interest, despite their unequal power in policy debates. To secure a greater respect for interdependence an ethical curb needs to be placed on unsustainable individualism in health debates. By advancing a more expansive and inclusive bioethic as a main foundation upon which problem solving within clinical, public health, and policy settings takes place (including those which utilize OH), humans may better respect co-species interdependence by situating human health within ecosystem health. In challenging the anthropocentrism and individualism of narrow, or traditional bioethics, the widespread practice of promoting human health at the detriment of non-human animals and the environment becomes less normal, and thus less acceptable. This is not to say that these older ethical frameworks do not have their place, but they cannot continue to rationalize the prioritization of human well-being alone. Rather than situating OH problem-solving within these narrow frameworks, a joined-up bioethic that works across health systems and helps people think beyond themselves is needed to guide conversations. This is because, although OH itself operates at a community or population-level, it must appeal to health and policy professionals and citizens who are enculturated within an individual approach to ethics. Although health services or systems are linked to primary and tertiary care provision, the ethical commitments of these cannot be seen as irrelevant to the OH agenda. The changes required by OH necessitates an emphasis on relationality across healthcare and policy, to make its claims pertinent. A more inclusive concept of justice—one that values the interests of non-human animals, the environment, and human health—should be made a core value in the process. With this praxis, humans will be better equipped to solve today’s most pressing global health problems in an ethically and scientifically sound and sustainable manner.

## Footnotes

1 In making an argument to protect the moral statuses of non-human animals and the environment, we assume that these species have moral status to some degree. To fully define the moral status of any given species requires a complex conversation debating the grounds of moral status, which is outside the scope of this paper. However, we offer a brief discussion on the moral status of non-human species in our section, “Justice in One Health,” to acknowledge the importance of this philosophical work both on its own and with respect to employing a One Health strategy.

2 We offer the Intuitive Equality Argument (Rowlands 1997) and the applications of the Difference Principle by Keliris-Thomas (2016) and Bell (2004) as further arguments for the inclusion of animals and the environment.

3 We acknowledge that achieving a truly neutral position will be difficult so long as humans are the arbitrators of their own justice, and indeed there is no other species which can assume this role. We also recognize that the institutions which utilize One Health as a strategy have their own agenda, which may overlap with what is moral to varying degrees. However, those applying the One Health strategy already share the belief that interdependence of humans, non-human animals, and the environment is key to a universal and sustainable health. It is our hope that this paper further elucidates a practical ethical framework which can be applied as part of the One Health strategy to global health’s most pressing issues.

4 Although we acknowledge that certain health organizations have policies endorsing disease control or eradication of farmed animals through measures like culling, the moral status of a species is determined not solely by its role to humans (i.e., farmed animals for meat vs wildlife to watch) but rather through a complex conversation about the grounds of moral status. This conversation includes at least in part an assessment of the species’ rights, interests, capabilities, capacities, and relationships to others. Thus, in this paper, we do not distinguish between bioethical approaches to various species, such as farmed animals versus wild animals, regarding One Health.
